# Therapeutic Targeting of the Proteolytic Enzymes

**DOI:** 10.3390/ijms24010521

**Published:** 2022-12-28

**Authors:** Frédéric Couture

**Affiliations:** 1 Pharmaceutical Sciences Department, TransBIOTech, Lévis, QC G6V 6Z3, Canada; frederic.couture@tbt.qc.ca; 2 Institute of Nutrition and Functional Foods, Laval University, Quebec, QC G1V 0A6, Canada; 3 Centre de Recherche du Centre Intégré de Santé et de Services Sociaux de Chaudière-Appalaches, Lévis, QC G6V 3Z1, Canada

## 1. Introduction

The processes regulating the generation of proteins from the early translation events to the final biologically active products are complex and tightly controlled. At the same time, the processes regulating the degradation are just as multifaceted, involving a wide variety of protease-activity-carrying enzymes that regulate proteolytic degradation in terms of space and time. Indeed, the notion that proteolysis is a kind of garbage disposal machinery allowing cells to degrade proteins so as to maintain general homeostasis was a commonly held view among the research community for a long time.

Over the last few decades, this perspective has evolved a with the numerous discoveries that have enlarged our view of the full landscape of proteolysis. We have gained a better understanding of the balance of endogenous inhibitors and the ability to decipher between targeted and untargeted proteolysis, with key impacts on the strict equilibrium between physiological and pro-pathological conditions. The complexity of these concepts becomes even greater when the different viewpoints are superimposed in order to take into consideration cell- and/or organ-specific redundancy. Moreover, the different circumstances leading to intracellular routing, which bring substrate(s) into the vicinity of the given protease(s), are vast and distinct scenarios that can impact on divergent biological functions, ranging from activation to inactivation.

Proteolysis is also exploited by virus and bacteria for pathogenesis and microbiocidal activity. Numerous types of virus exploit the proteases from host cells to perform viral protein processing, including the spike proteins of coronaviruses, such as SARS-CoV-2, and hemagglutinins from myxoviruses, such as those involved in influenza infection. On the other hand, some bacteria use host proteases to activate their toxins and yield their cytotoxic effects on eucaryotic cells, such as anthrax, diphtheria, or clostridial toxins. The bacteria themselves use coordinated proteolysis to generate bioactive antimicrobial peptides with a startling proteolytic resistance that display a particular lasso-shaped ring.

Thus, the field of proteolysis has grown rapidly and attracted a great deal of attention with regard to the therapeutic potential of several proteases, resulting in the generation of numerous inhibitors, of which several are now on the market. However, it is clear that this increasing interest also continues to broaden the foreground of questions that must be addressed in order to better understand how and to what extent a given proteolytic axis can be targeted in a given pathological condition and how it will affect physiology. Only with these answers in hand will it be possible to optimize the rational design of therapeutic strategies targeting currently unexplored proteases.

This Special Issue on the “Therapeutic Targeting of the Proteolytic Enzymes” covers this spectrum of questions in regard to proteases and their biological activity in order to better assess their pharmacological potential.

## 2. Endoproteolysis: From Substrate Redundancy to SARS-CoV-2 Infection

In this review, Gary Thomas provides a historical analysis of the pioneering works that led to the discovery of the proprotein convertase Furin, its involvement in diseases, and efforts to develop compounds that can inhibit this enzyme [1]. Furin, together with other members of the proprotein convertase family, belongs to the class of endoproteolytic enzymes with very precise cleavage site at dibasic residues involved in the biological processing of proteins termed proproteins during their trafficking within cells. Although very similar in terms of their biological activities and cleavage site preferences, these proteases’ activities have distinctive redundancy levels due to their distinct intracellular localizations and expression patterns in different cell types and tissues. This distinction has long been the critical element jeopardizing their exploitation as pharmacological targets. As of today, numerous studies have highlighted the capacity to target certain non-redundant functions of certain key PCs, such as Furin, in pathological conditions, while preserving the endogenous activity by either local delivery or local redundancy due to other PCs. This provides key evidence for studies such as that published by Coppola et al. in this Special Issue, where the authors demonstrated the distinct redundancy levels of Furin and other PCs at processing insulin receptors in the liver hepatocytes and pancreas β cells using CRISPR-generated cell-specific knockout models in mice [2]. Interestingly, they demonstrated that IR processing is unaffected in Furin-deficient hepatocytes but is blocked in Furin-deficient pancreatic β cells, with the development of only slight glucose intolerance over time.

This manuscript highlights that the processing of substrates by members of the proprotein convertases, such as Furin, can be more tissue-dependent than expected from certain cell-based studies, which should be acknowledged when considering these proteases as targets. This is notably the case for SARS-CoV-2 infection, which necessitates cleavage by host proteases such as Furin and TMPRSS2. These proteases have attracted a great deal of attention during the recent pandemic, since the targeting of host, rather than viral, targets may represent an important advantage that can be exploited to prevent the occurrence of resistance. Such a perspective is reviewed by Wettstein and colleagues in a comprehensive review of TMPRSS2’s involvement in the entry of SARS-CoV-2 into cells during infection, as well as the clinical development of inhibitory compounds [3].

## 3. Biological Protease Inhibitors: A Strict Balance Covering a Large Range of Activities, from Inflammation to Bacterial Competition 

Proteolysis represents a finely tuned equilibrium between biologically active proteinases and the vast variety of inhibitors that cells generate in order to regulate the degree of activity, sometimes in the form of activation cascades, which can occur in a local manner. An important example of such a mechanism is coagulation, which can be manipulated by several classes of protease inhibitors. In a review published in this Special Issue, Yamada and Asakura summarize the different therapeutical avenues for disseminated intravascular coagulation, which is a rare and complex procedure used to treat blood coagulation conditions [4]. They cover the different options for therapies and the underlying mechanisms and diagnostic markers. 

In their research article, Yoshida and colleagues investigated the involvement of the serine protease inhibitor alpha1-antitrypsin (A1AT), generated by trophoblasts cells, and its impact on the syncytialization process [5]. They showed that this protein inhibitor triggers the activation of the p38 MAPK pathway, which may mediate abnormal placental formation. The optimal expression of A1AT in trophoblasts thus appears to be critical for placental syncytialization and inflammatory responses, and villous A1AT expression may play a role in some early abortion- as well as pregnancy-associated hypertensive disorders.

Bacteria utilize an intriguing post-translational process to generate modified antimicrobial peptides in order to control the growth of other bacteria in their environment. Among them, Microcin J25 is one of the most well-known, notably for its lasso structure, generated by the serial actions of bacterial proteinase and cyclase in generating isopeptide bonds, rendering these compounds extremely resistant to thermal or proteolytic degradation. Malik and colleagues used this backbone to graft binding motifs onto the loop region of this lasso peptide in order to yield novel Microcin J25 variants that are able to bind to and affect the activity of the Clp serine proteases [6]. Their work revealed novel kinds of ClpP binders that cannot be degraded by Clp proteases and may serve as a further scaffold for novel antibiotics.

## 4. Concluding Remarks

The landscape of the field has radically evolved since the stem works that aimed to isolate activity-carrying fractions and further characterize them so as to identify proteases. As of today, several key proteases have already been identified, and the substrate and/or inhibitor molecular determinants have been documented for a good proportion of them. This information has always been valuable for medicinal chemists aiming to rationally design inhibitors with a maximized specificity and selectivity, notably through the identification of allosteric sites that can be used to build out-of-catalytic site inhibitory molecules. These efforts have clearly yielded significant advances with success in clinical trials, enabling new compounds to reach patients. Henceforth, with the democratization of novel technologies, such as efficient RNA interference (RNAi) strategies and the delivery of targeted protein degraders (PROTACs), both of which are becoming increasingly available as orally administered therapies, the targeting of currently hard-to-target proteases has become easier and will likely accelerate further development in this field.
